# 共价有机框架色谱固定相研究新进展

**DOI:** 10.3724/SP.J.1123.2026.01026

**Published:** 2026-11-08

**Authors:** Kaixing LUO, Ying SUN, Qiuting ZHANG, Lusha WU, Jiamin SONG, Yanhui ZHONG, Zian LIN

**Affiliations:** 福州大学化学学院，食品安全与生物分析教育部重点实验室，福建省食品安全分析与 检测重点实验室，福建 福州 350108; Ministry of Education Key Laboratory of Analytical Science for Food Safety and Biology，Fujian Provincial Key Laboratory of Analysis and Detection Technology for Food Safety，College of Chemistry，Fuzhou University，Fuzhou 350108，China

**Keywords:** 共价有机框架, 固定相, 液相色谱, 气相色谱, covalent organic frameworks （COFs）, stationary phase, liquid chromatography （LC）, gas chromatography （GC）

## Abstract

色谱是一种重要的分离分析技术，广泛应用于环境监测、生命科学、食品安全和药物分析等领域。作为色谱分离技术的核心，色谱固定相对分离性能有着决定性作用。以硅胶为基质的固定相尽管已被广泛应用于色谱分离，但硅胶基质存在制备工艺复杂、传质阻力大、pH适用范围窄等缺点，严重阻碍其深入应用。因此，开发兼具高化学稳定性和高分离性能的新型固定相以满足不同场景的分离需求，始终是色谱分析领域的研究热点。共价有机框架（COFs）作为一类新兴的多孔晶态材料，具有比表面积大、孔道结构规整、高化学/热稳定性等显著优势，不仅有望克服上述问题，还可通过对孔道功能化修饰实现对目标分析物的精准识别，因而被认为是构建高性能色谱固定相的理想材料。本文系统述评了近两年来COFs在气相色谱与液相色谱固定相应用方面的研究进展，重点围绕COFs材料的结构设计与性能调控，阐述其在有机化合物广谱分离、位置异构体分离以及手性化合物拆分等方面的应用与分离机制。在此基础上，结合当前研究现状与存在问题，对COFs基色谱固定相的发展方向与应用前景进行了分析与展望。

## Abstract (en)

Chromatography is an important separation technique that has been widely applied in environmental monitoring，life sciences，food safety，and pharmaceutical analysis. As the core of chromatographic separation technologies，the stationary phase plays a decisive role in separation performance. Although silica-based stationary phases have been extensively used in chromatographic separations，their further application is severely limited by inherent drawbacks，such as complicated preparation procedures，high mass transfer resistance，and narrow applicable pH ranges. Therefore，the development of novel stationary phases that simultaneously exhibit high chemical stability and excellent separation performance to meet diverse separation requirements has remained a long-standing research focus in the field of chromatographic analysis. Covalent organic frameworks （COFs），as an emerging class of porous crystalline materials，possess distinctive advantages such as large specific surface areas，well-defined and ordered pore structures，and high chemical and thermal stability. These characteristics not only enable COFs to overcome the limitations of conventional chromatographic materials，including slow mass transfer and narrow applicable pH ranges，but also allow for precise recognition and selective separation of target analytes through rational functionalization of their pore structures. Consequently，COFs have been widely recognized as highly promising candidates for the construction of high-performance chromatographic stationary phases. This review systematically summarizes recent advances in the application of covalent organic framework （COF）-based stationary phases for gas chromatography （GC） and liquid chromatography （LC） over the past two years. Emphasis is placed on the rational structural design and performance modulation of COF materials，with particular attention to their applications in the broad-spectrum separation of organic compounds，positional isomer discrimination，and enantioseparation. The underlying separation mechanisms and structure-property relationships are discussed in detail. In light of the current research status and existing challenges，future development directions and potential applications of COF-based chromatographic stationary phases are further discussed，aiming to provide insights for the continued advancement of this emerging field.

色谱技术是生物、医药、食品和精细化工等国民经济重要行业的共性技术，在大规模物质分离纯化方面的重要性日益增加，尤其在生物制药领域，色谱技术几乎是生物制药分离纯化的唯一手段，高纯度、高活性的生物制品制造基本都依赖于色谱分离技术^［1-3］^。

传统的色谱固定相主要由硅胶颗粒构成，因其易于修饰且操作简便、快捷，而获得了广泛的商业化应用。然而，硅胶基质的固有缺陷限制了其进一步应用：具有狭窄的pH耐受性（仅适用于pH=2~8），尤其在强碱性条件（pH>8）下易发生骨架水解；表面残留的活性硅羟基可能引发极性化合物的不可逆吸附，导致峰拖尾。因此，开发兼具高化学稳定性和高分离性能的色谱固定相已成为色谱领域的研究热点。有序多孔材料如金属有机框架（metal organic frameworks，MOFs）、共价有机框架（covalent organic frameworks，COFs）、多孔有机笼（porous organic cages，POCs）等凭借其均匀的孔径、规整的孔隙排列、可调的骨架结构以及高比表面积，在色谱分离领域展现出了卓越的应用潜力^［4-6］^。其中，COFs是由轻质元素通过共价键连接组成的结晶性有机框架材料，它们的结晶性源于结构单元之间的动态共价键，这些键能够在合成过程中实现自我修复和纠错，从而形成高度稳定的结构^［7］^。与传统的硅基固定相材料相比，COFs具有诸多优势，如低密度、强疏水性、强*π-π*相互作用和高载碳能力^［8，9］^。最重要的是，其表面化学特性可根据特定分析物的需求进行定向调控，这一优势促使越来越多的色谱工作者致力于开发新型的COFs材料固定相。本文主要述评了近两年COFs在高效液相色谱（HPLC）和气相色谱（GC）领域的应用，为COFs在分离领域的进一步探索提供思路。

## 1 基于共价有机框架的色谱固定相制备

### 1.1 COFs涂层气相色谱固定相

传统气相色谱固定相的发展长期受限于分离选择性、可调控性不足以及涂覆均匀性差等核心难题。COFs作为一类具有可设计孔道结构、高比表面积和优异热稳定性的结晶性多孔材料，为解决这些难题提供了新思路。Deng等^［10］^报道了一种用于制备COF涂层开管毛细管柱的通用方法：以1，3，5-三（4-氨基苯基）苯（Tab）和2，5-二羟基-1，4-苯二甲醛（DHTA）为单体，先在毛细管内壁形成均匀的非晶聚合物层，再将其转化为高结晶度的Tab-DHTA COF层，成功制备了Tab-DHTA涂层毛细管柱（图1）。该柱对多种位置异构体表现出良好的分离效果。该策略不仅实现了高结晶COF层在毛细管内壁上的均匀涂覆，还为制备高选择性、高均匀性的COF涂层毛细管柱提供了通用途径。随后，Zeng等^［11］^系统探究了COF结晶度这一关键结构参数对分离性能的影响，他们以1，4-二氰基苯（DCB）为构筑单元，合成了3种结晶度不同的共价三嗪框架材料（CTF-DCB-1、CTF-DCB-2和CTF-DCB-3），发现高结晶度的CTF-DCB-1凭借规整的孔道结构和最小的扩散阻力，实现了对苯系异构体的高效分离；而低结晶度材料则因孔道无序和扩散阻力大导致分离性能下降。该研究表明，在获得均匀涂层并优化晶体形态的基础上，进一步提升COF的结晶度是构建有序扩散路径、实现高效稳定分离的关键策略。近期，Deng等^［12］^报道了一种通过调控二维COF层间堆叠结构以提升其气相色谱分离性能的有效策略。该研究以5，10，15，20-四（4-氨基苯基）-21*H*，23*H*-卟啉（Tph）与DHTA为单体合成的Tph-DHTA为模型体系，研究表明，通过改变溶剂体系，可将COF的层间堆叠结构从常见的重叠-AA（e-Tph-DHTA）调控为滑移-AA（s-Tph-DHTA），显著提高了C8芳香烃异构体的分离分辨率和速度。该研究展示了调节COF层间堆叠结构在提升分离性能方面的巨大潜力。此外，COF的功能化是制备高分辨率色谱固定相的关键策略之一。Deng等^［13］^在前期研究基础上，引入含甲氧基取代基的2，5-二甲氧基对苯二甲醛（DMTA）作为第三单体，将其掺入Tab-DHTA体系中，成功构建了三组分COF材料Tab-DHTA-（DMTA）_x%_。通过调控DMTA的掺入比例，实现了对COF体系中氢键作用与范德华力相互作用的有效平衡。研究结果表明，该COF在保持Tab-DHTA对多种位置异构体良好分离能力的同时，显著提升了分离速率和柱效，表现出更优异的色谱分离性能。总体而言，COF涂层气相色谱固定相的研究正在向结构有序化、功能精细化和性能高效化方向发展，为复杂样品体系的高效分离分析奠定了坚实的材料基础。

**图1:**
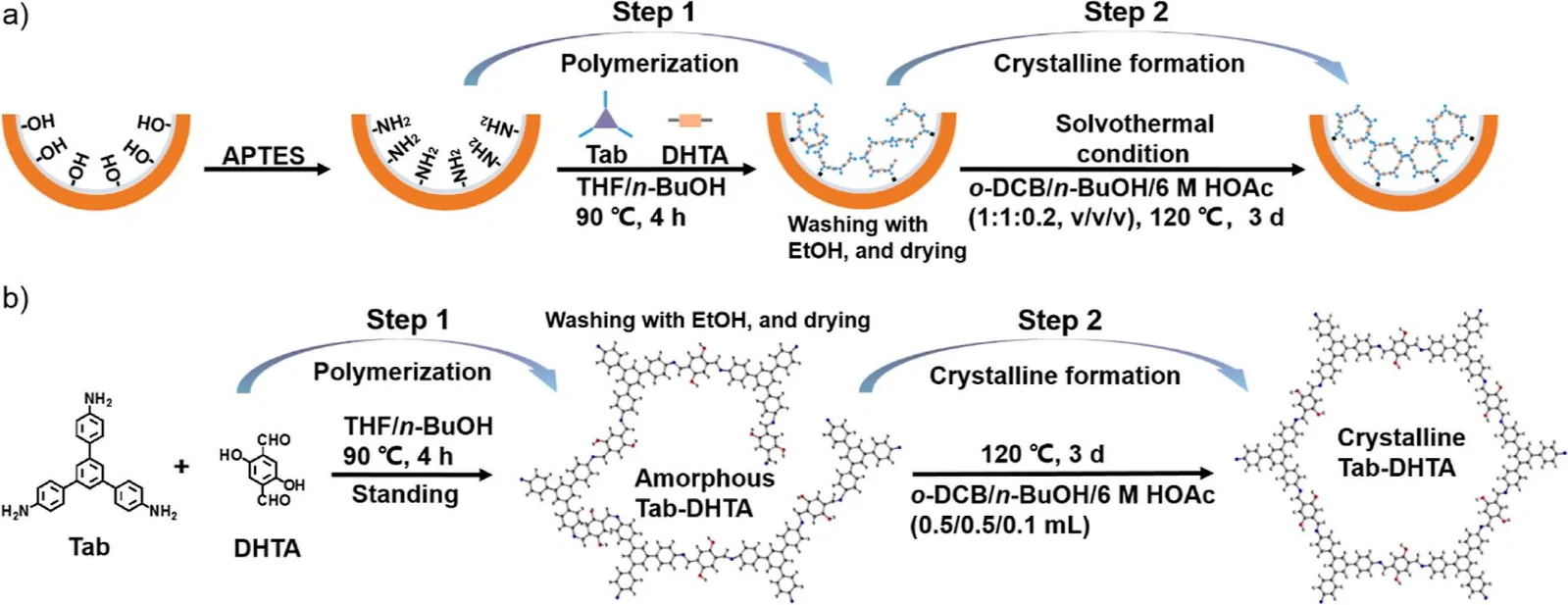
（a）制备Tab-DHTA结合开管毛细管柱的两步法示意图；（b） Tab-DHTA COF的两步合成示意图^［10］^

### 1.2 SiO_2_@COFs复合材料液相色谱固定相

COFs具有孔道结构规整、比表面积高以及化学稳定性优异等特性，其在液相色谱分离领域具有广阔的应用前景。尽管如此，目前已报道的大多数COFs因不规则形状和较宽的粒径尺寸分布难以直接作为色谱固定相，这成为COFs在HPLC固定相应用的瓶颈。针对上述问题，研究者提出了复合化与功能化的解决策略，其中以二氧化硅（SiO_2_）微球为载体构建的COF@SiO_2_复合材料已成为当前主流的固定相研究方向之一。2022年，Yuan等^［14］^创新性地将含手性冠醚基团的COFs通过“网包法”固载于SiO_2_表面，利用冠醚在孔道内的周期性排列实现多类外消旋化合物的对映选择性识别，并且所获得的两种手性固定相具有较高的稳定性，可同时应用于GC和HPLC的分离中（图2）。随后，Chen等^［15］^提出一种多手性协同的设计思路，通过分步修饰，将甲基化环糊精（MCD）与谷胱甘肽（GSH）引入非手性COFs骨架中，构建了多手性COFs，并将其与SiO_2_结合得到GSH-MCD-COF@SiO_2_固定相（图3）。该材料中MCD善于识别手性醇与胺类化合物，而GSH则对氨基酸和有机酸具有强选择性，两者的协同作用在COFs孔道内形成了受限的手性微环境，显著拓宽了GSH-MCD-COF@SiO_2_固定相分离手性对映体的范围。此外，为克服COFs材料疏水性问题，Xu等^［16］^以2，4，6-三（4-氨基苯基）-1，3，5-三嗪（TAPT）与2，5-二溴苯-1，4-二甲醛（TaBr_2_）为单元合成了TAPT-TaBr_2_ COF并修饰于SiO_2_表面，再通过自由基聚合在其表面接枝亲水性纳米水凝胶（hydrophilic nanohydrogel，NHG），制备出混合模式固定相NHG-COF@SiO_2_。NHG的引入不仅有效调节了材料表面亲疏水平衡，还实现了对不同极性化合物及位置异构体的高效分离。以上研究表明，针对COFs粉体形貌不规则、粒径分布宽及难以直接装柱等问题，构建COF@SiO_2_复合材料已成为提升其色谱适用性的有效途径，为COFs在HPLC固定相领域的深入应用奠定了坚实基础。

**图2:**
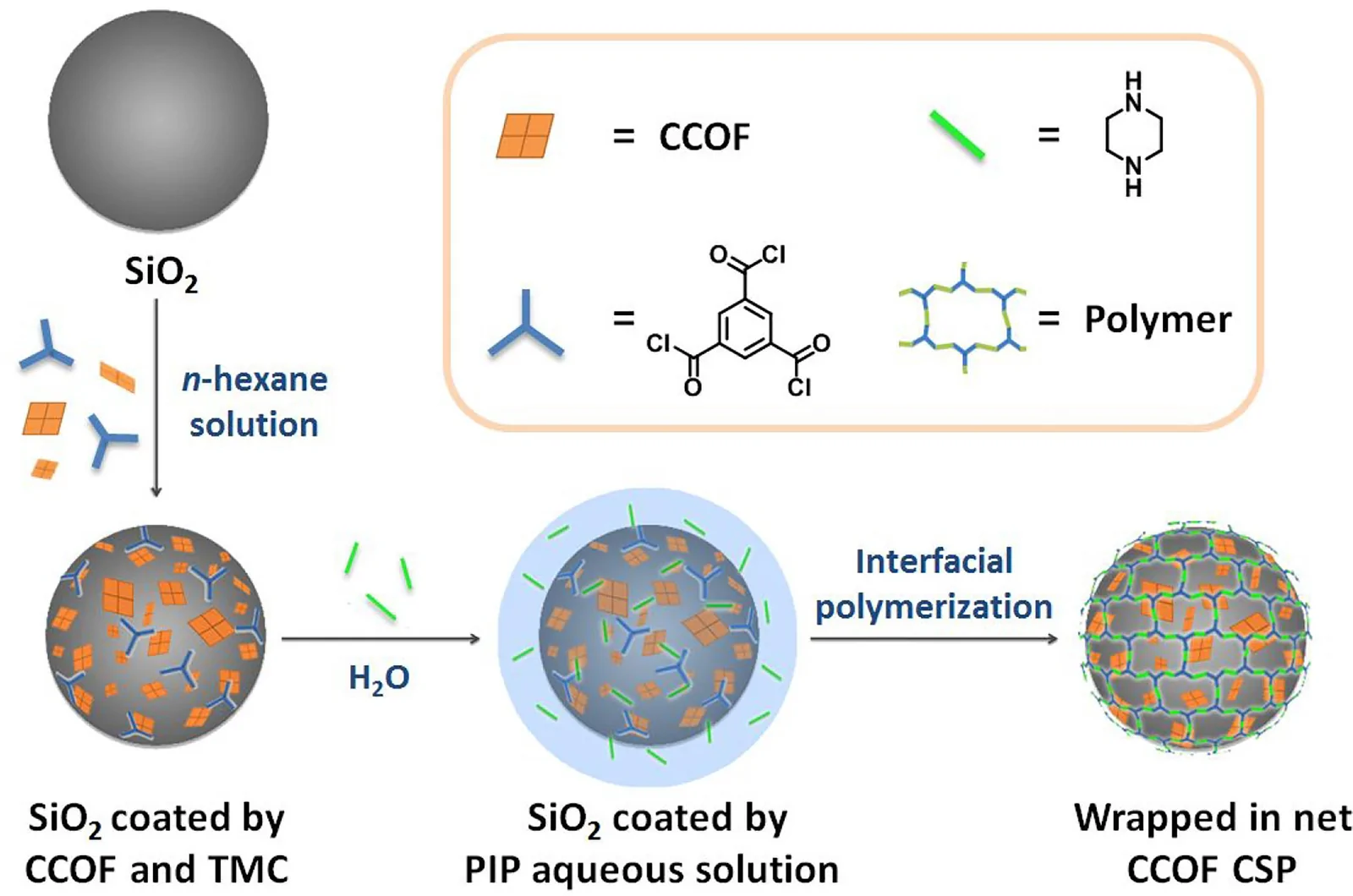
“网包法”COFs手性固定相的制备程序^［14］^

**图3:**
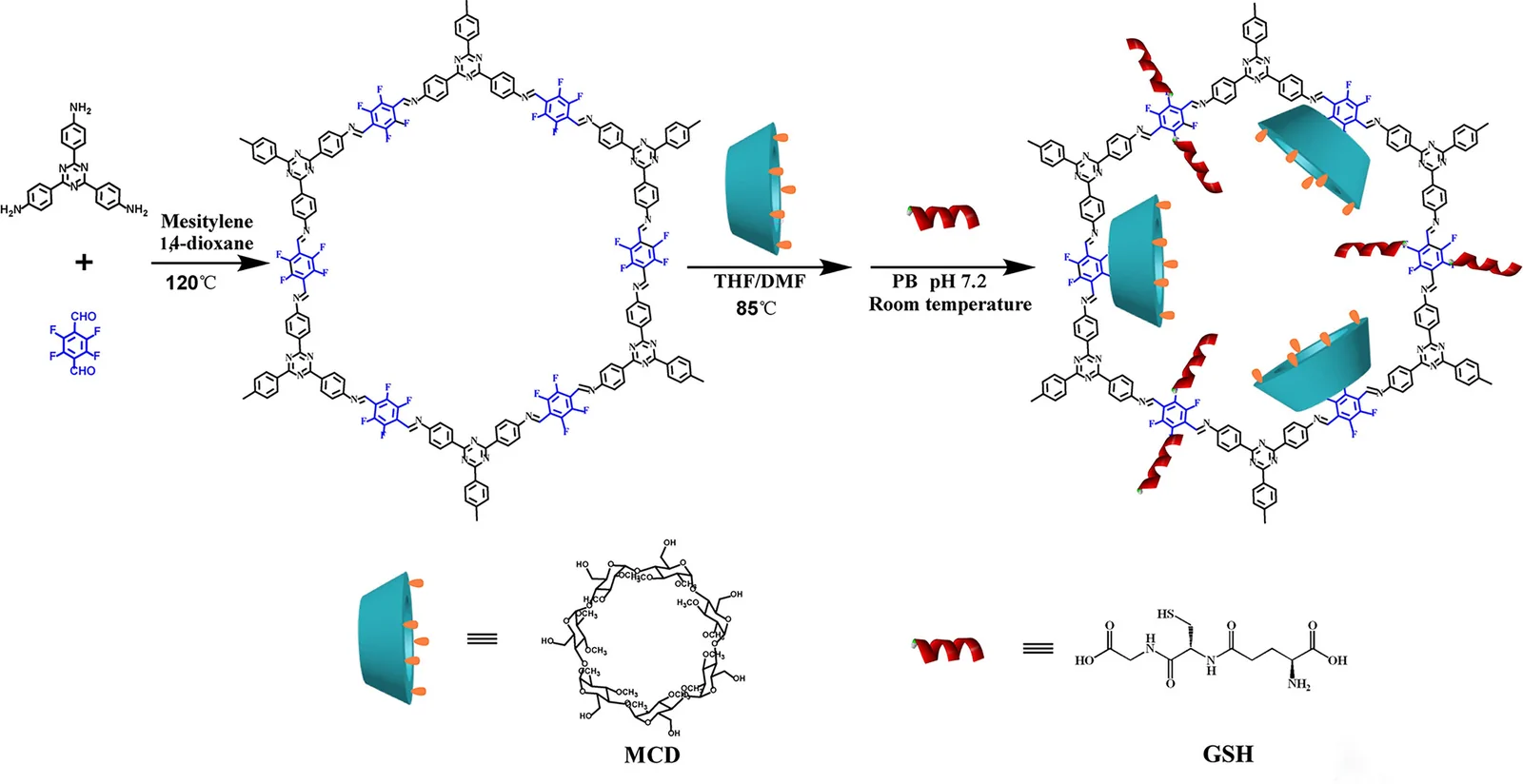
双手性COF（GSH-MCD COF）制备示意图^［15］^

### 1.3 COFs液相色谱固定相

随着COF合成技术的发展，球形COFs作为液相色谱固定相的研究近年来取得显著进展，其均一粒径与功能化特性为高效分离提供了新途径。2019年，Ma等^［17］^在室温下可控合成了从纳米到微米尺度的均匀球形COFs，所制得的不同粒径的球形COFs具有超高的比表面积、良好的结晶度和化学/热稳定性，这使得选择COFs直接作为HPLC固定相成为可能。Zheng等^［18］^以1，3，5-三（4-氨基苯基）苯（TAPB）和2，5-二乙烯基对苯二醛为单体，乙酸溶液为催化剂，室温下可控合成微米级球形COFs并首次用于HPLC分离。结果表明，该球形COFs填充柱能够高效分离不同种类的疏水性小分子，还成功应用于蛋白质（牛血清白蛋白）酶解液的分离。上述研究表明，球形COFs的形貌调控及其在色谱领域的应用已取得突破性进展。研究者通过室温可控合成技术，有效解决了COFs材料从无定形粉末向均一微米球转变的难题，赋予了固定相优异的装填性能与传质效率，实现了对复杂样品的高效分离。

近年来，单晶共价有机框架（single-crystal covalent organic frameworks，SCOF）的精准构筑技术不断取得突破，推动了其在色谱分离应用领域的快速发展。相较于传统多晶材料的结构无序性，SCOFs依托长程有序的分子排列和可精确调控的孔道拓扑结构，在分子识别与分离研究中表现出显著优势。Zheng等^［19］^以对苯二甲醛与四（4-氨基苯基）甲烷为单体，采用乙酸作为催化剂，苯胺作为成核抑制剂，在室温条件下合成了SCOF-300，并将其用于HPLC固定相的制备（图4a）。研究发现，单晶结构显著提升了COFs的比表面积利用率与分子识别能力。其高度有序、规整的孔道结构为分子吸附与传质提供了均一通道，使材料在复杂有机体系中表现出优异的选择性与分离性能，尤其能够实现对理化性质高度相似的位置异构体的高效分离。在此基础上，Zhang等^［20］^进一步系统研究了单晶3D COFs在疏水小分子及临床药物的广谱分离中的应用潜力。结果表明，单晶3D COFs具有互穿孔道、高比表面积及出色化学稳定性，使得该材料在疏水分子体系中表现出优异的相互作用与识别能力，为研究复杂体系中3D COFs的选择性分离与分子识别行为提供了新的可能。此外，Wu等^［21］^基于四（4-醛苯基）甲烷（TFM）与PDA在室温下合成了胶囊状SCOFs，并直接用于构建液相固定相（图4b）。该材料具有独特的胶囊型形貌与高度单分散特性，其规整的孔隙结构和显著的疏水性使其在分子识别与选择性分离中展现出卓越性能。研究表明，胶囊状SCOFs兼具高柱效与优异的结构稳定性，不仅能够高效识别疏水分子，还可区分理化性质相似的多种位置异构体。上述研究从形貌调控、多晶到单晶结构优化以及功能基团定向修饰等多个层面，持续推进了COFs材料在液相色谱固定相领域的结构精细化与性能提升，为新型高性能色谱固定相的构筑提供了重要理论与实验依据。

**图4:**
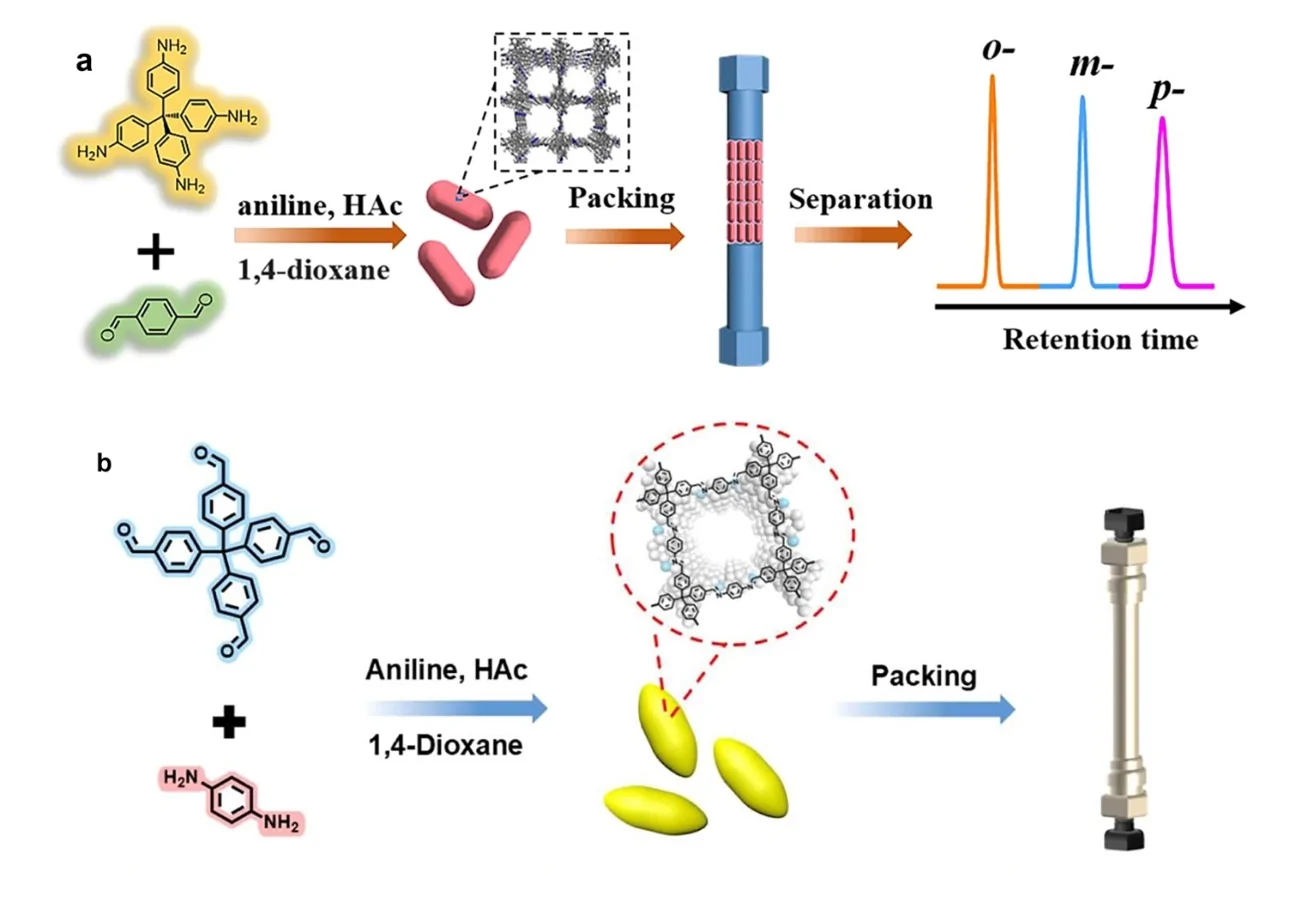
（a）单晶COF-300填充柱的合成^［19］^；（b）胶囊型SCOFs填充柱的合成^［21］^ SCOFs： single-crystal covalent organic frameworks.

## 2 共价有机框架材料在色谱分离中的应用

### 2.1 有机化合物的广谱分离

在复杂样品分离中，基于COFs的色谱固定相已广泛应用于药物分析和环境污染物监测等领域，展现出优异的选择性和高效性。Zhang等^［20］^将单晶3D COFs直接作为HPLC的固定相，用于疏水小分子的广谱分离（图5）。结果表明，该填充柱不仅能够高效分离取代苯类、卤代苯类、卤代硝基苯类、芳香胺类、芳香烃以及邻苯二甲酸酯（PAEs），而且还能实现对物理和化学性质非常相似的化合物（如苊和苊烯）以及药物（如卡马西平和卡马西平-10，11-环氧化合物）的基线分离。其中，PAEs色谱柱效达到17 303~24 255 plates/m，苊和苊烯、卡马西平和卡马西平-10，11-环氧化合物的分离度（*R*_s_）分别为1.7和2.2。与商用C18色谱柱相比，单晶3D COFs固定相依托其较高的比表面积和有序三维孔道结构，以及芳香骨架所带来的疏水作用与*π-π*相互作用的协同效应，表现出更高的分离效率和选择性，在疏水芳香族小分子的高效分离方面展现出明显优于传统C18固定相的应用潜力。Xu等^［16］^制备的NHG-COF@SiO_2_色谱柱表现出令人满意的分离能力，在单根色谱柱上成功实现了多环芳烃、烷基苯、苯酮、核苷碱基和水溶性维生素等不同极性的小分子化合物的分离。最后，还将NHG-COF@SiO_2_用于实际水样的检测当中，有效检测出了水样（中国武汉东湖）中的多环芳烃。以上结果表明，基于COFs的新型固定相在有机化合物的广谱分离领域具有巨大的应用潜力。

**图5:**
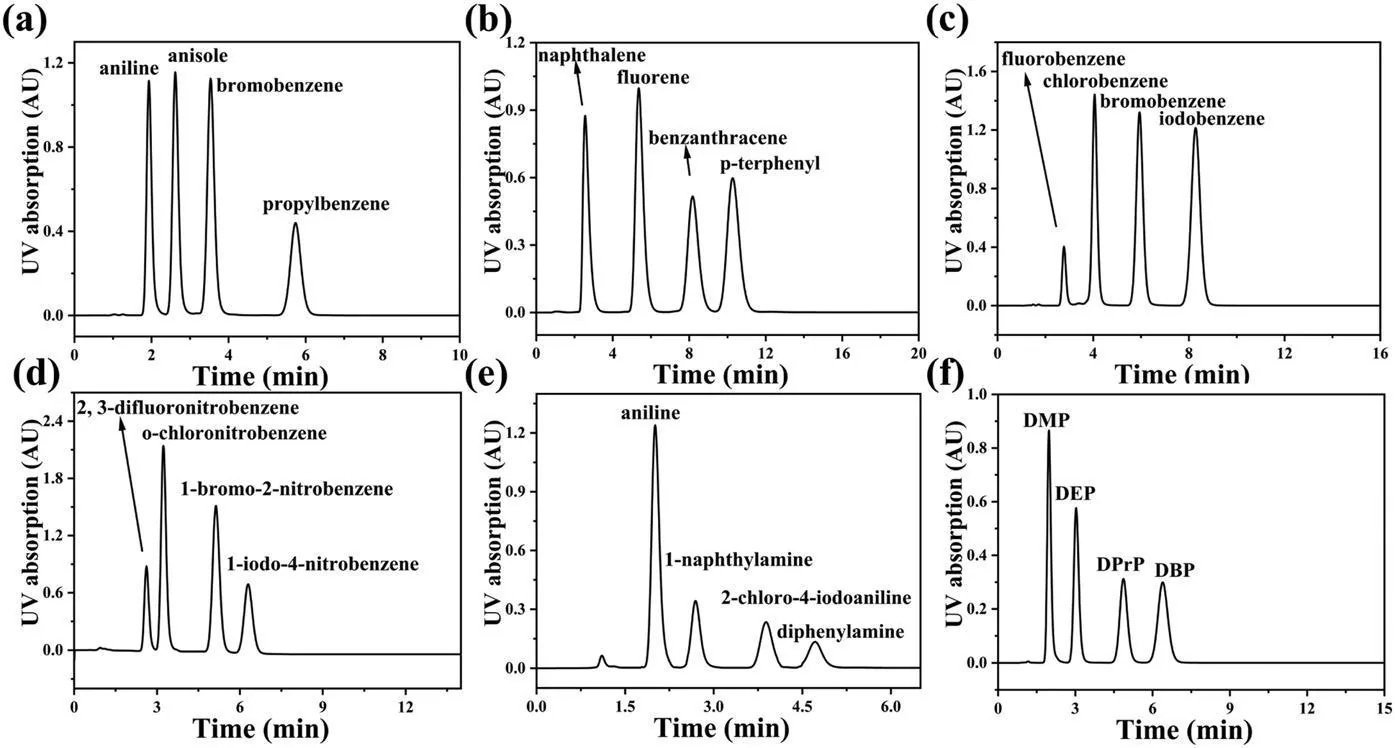
疏水化合物在单晶3D COFs填充柱上的色谱分离性能^［20］^

### 2.2 位置异构体的分离

位置异构体是分子式相同、碳骨架相同，但官能团、取代基或特征结构（如双键、三键）在碳链或（环上）的位置不同的一类构造异构体，其物理和化学性质非常相似，分离极具挑战性。Zheng等^［19］^将SCOF-300用作固定相，成功实现了多种位置异构体的高效分离。该SCOF-300填充柱对硝基苯胺、二氯苯、二溴苯、二碘苯、二乙苯、氯硝基苯、溴硝基苯以及碘硝基苯等8种异构体展现出优异的分离性能。其中，*m-/p-*二碘苯的*R*_s_达到4.45，*o-/m-*二碘苯为2.53，分离效果明显优于商用C18色谱柱及多晶COF-300填充柱，突显了该固定相在异构体分离中的潜力与优势^［19］^。Deng等^［10］^所制备的Tab-DHTA涂层毛细管柱对乙苯、二甲苯、二氯苯、氯甲苯、蒎烯、1，3-二氯丙烯和丙苯等异构体具有良好的分离度（>1.5），且柱效最高可达39 663 plates/m。该研究还评估了毛细管柱的日内与日间重复性。以二氯苯为分析物，重复进样5次（*n*=5），其保留时间、峰面积、峰高及半峰宽的相对标准偏差（RSD）分别为0.2%~0.6%、0.5%~1.1%、0.5%~1.4%和1.2%~2.4%，显示该色谱柱具有优异的重复性和稳定性。Wu等^［21］^在室温下合成了胶囊型SCOFs，并用于物理化学性质相似的异构体的基线分离，通过系统对比商用C18色谱柱和多晶COF柱，进一步评估其分离性能。对于二甲苯异构体，胶囊型SCOFs柱可实现基线分离，柱效高达50 700~60 600 plates/m，而商用C18柱即使优化流动相条件仍无法实现有效分离。对于对三联苯、萘酚异构体、二乙基己烯雌酚异构体及甲氧基补骨脂素异构体等，胶囊型SCOFs 柱均实现了*R*_s_>1.5的分离效果，而C18柱及多晶COF柱分离能力明显不足。此外，在卤代甲苯、卤代苯甲醚及卤代三氟甲基苯等系列位置异构体分离中，胶囊型SCOFs固定相表现出更高的分离因子与分离度，部分异构体的*R*_s_值可达2.95和3.13，而C18柱选择性较低（图6）。结果表明，胶囊型SCOFs填充柱对多类位置异构体均表现出良好的保留与分离能力，而在相同色谱条件下，商用C18柱对上述化合物保留不足，难以实现有效分离。本研究促进了具有独特结构的SCOFs作为色谱分析潜在固定相的发展，扩大了SCOFs的应用范围。Deng等^［13］^制备的三组分COF固定相Tab-DHTA-（DMTA）_x%_显著提升了对乙基戊酸酯异构体的分离能力。与母体Tab-DHTA固定相无法实现关键异构体基线分离相比，三组分固定相可实现4种乙基戊酸酯异构体的完全基线分离，*R*_s_>1.5，柱效达到23 021~29 796 plates/m。值得注意的是，该固定相在提升柱效和缩短分析时间的同时，并未削弱其对二氯苯、氯甲苯、蒎烯、1，3-二氯丙烯及丙基苯等多类结构异构体的分离能力，仍保持*R*_s_=1.6~5.1及7 243~27 328 plates/m的高柱效水平。总体而言，该多组分构筑策略为COF固定相的可控官能化提供了新思路，通过精确调节孔道内相互作用类型与强度，实现了高柱效与高选择性的协同优化。

**图6:**
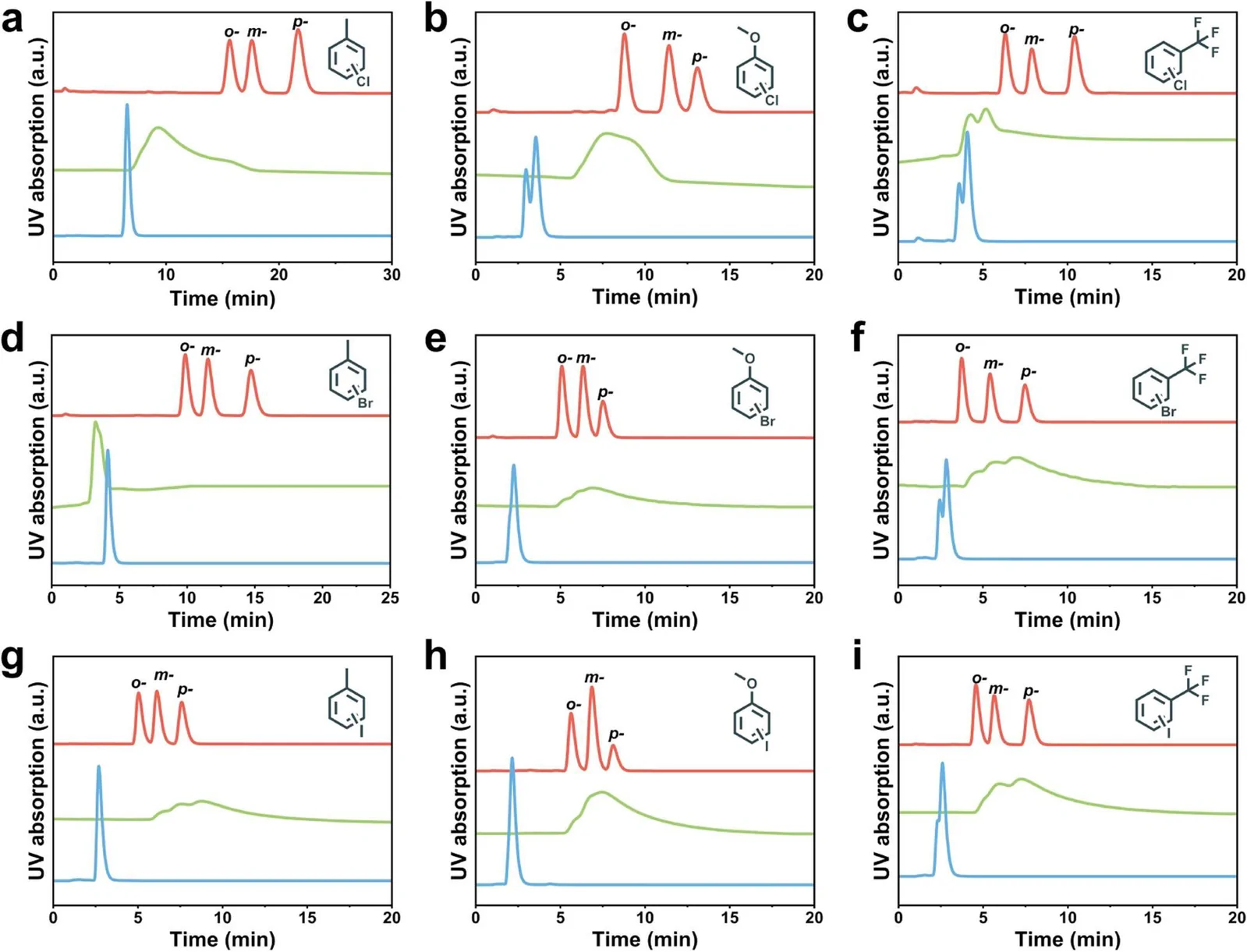
在胶囊型SCOFs填充柱（红色）、多晶COF填充柱（绿色）和商用C18柱（蓝色）上分离位置异构体^［21］^ a. chlorotoluene isomers； b. bromotoluene isomers； c. iodotoluene isomers； d. chloroanisole isomers； e. bromoanisole isomers； f. iodoanisole isomers； g. chlorobenzotrifluoride isomers； h. bromobenzotrifluoride isomers； i. iodobenzotrifluoride isomers.

### 2.3 手性化合物的分离

手性对映体通常具有不同的药理学、毒理学和生理学效应，但其在物理和化学性质上几乎相同，分离手性化合物具有很大的挑战性。Yuan等^［14］^制备的冠醚基团COF手性色谱柱对多种外消旋体（包括谷氨酸、乳酸乙酯、苯丙氨酸和2-甲基戊醛等）也具有较好的分离性能，其*R*_s_最大可达4.56。Chen等^［15］^将合成的GSH-MCD COF用于手性对映体分离。结果表明，该COF固定相对多种手性芳香醇、芳香酸、酰胺、氨基酸和有机酸具有良好的分离效果。在色谱图中这些化合物均展现出了清晰的分离峰，并且获得的分离因子（*α*）和*R*_s_相较于商用色谱柱均有所提升。这一发现有力验证了协同多手性COF在分离和识别性能上的优越性。由此可见，手性COF作为手性色谱固定相在对映异构体分离方面具有广阔的应用前景。

## 3 总结与展望

COFs作为新型色谱固定相，凭借其可设计孔道结构和多样化相互作用模式（如疏水、亲水、氢键、*π-π*及静电作用），在分离亲疏水化合物、异构体及对映体方面展现出显著优势。然而，其在色谱分离领域的进一步应用仍面临若干挑战。（1）COFs的合成与精准功能化能力仍受限制。目前，COFs的合成过程往往条件苛刻、可控性不足，尤其是在晶体形貌、粒径均一性及结晶度调控方面仍存在挑战，这在一定程度上影响了其重复性与色谱分离性能。同时，功能基团的引入多依赖后修饰或前驱体设计的策略，难以在孔道内实现真正意义上的精准、可预测调控。（2）COFs色谱固定相的应用范围仍有待拓展。现有研究主要集中于有机小分子、位置异构体及部分手性化合物的分离，在生物大分子、复杂天然产物、环境样品及工业体系等真实复杂样品中的系统研究仍较为有限。此外，多数工作仍停留在实验室验证阶段，与商品化色谱柱在长期稳定性、批次一致性及实际样品适用性方面的系统比较不足。

综上所述，COFs作为新型色谱固定相在结构可设计性和分离选择性方面展现出独特优势，但其进一步发展仍受到合成可控性与应用深度不足的制约。未来研究需在优化合成策略、实现精准功能化的同时，持续拓展其在复杂真实样品和实际分析场景中的应用。通过加强对稳定性、重现性及规模化可行性的系统评估，有望推动COFs色谱固定相从基础研究走向更广泛的实际应用，为色谱分离技术的发展提供新的研究思路。
